# Neuroprosthetics for Auricular Muscles: Neural Networks and Clinical Aspects

**DOI:** 10.3389/fneur.2017.00752

**Published:** 2018-01-16

**Authors:** Mikee Liugan, Ming Zhang, Yusuf Ozgur Cakmak

**Affiliations:** ^1^Department of Anatomy, University of Otago, Dunedin, New Zealand

**Keywords:** extrinsic, intrinsic, auricular muscles, neuroprosthetics, pinna, facial

## Abstract

The mammalian external ear houses extrinsic and intrinsic auricular muscles. There are three extrinsic auricular muscles—the posterior, superior, and anterior auricular muscles—and six intrinsic muscles—the helicis major and minor, tragicus, anti-tragicus, transverse and oblique muscles. These muscles have been considered vestigial in humans. However, numerous therapeutic and diagnostic wearable devices are designed to monitor and alleviate the symptoms of neurological disorders, brainstem injuries, emotional states, and auditory functions, by making use of the neural networks of the auricular muscles and their locations, which are easily accessible for ergonomic wearable biomedical devices. They can also serve as a bio-controller of human neuroprosthetics. The functionality of these auricular muscles remains elusive and requires further experimentation for a more in-depth understanding of their anatomy. The aims of this review are (1) to provide a detailed account of the neural networks of the extrinsic and intrinsic auricular muscles, (2) to describe diagnostic and therapeutic functions of these muscles as demonstrated in the current literature, and (3) to outline existing and potential neuroprosthetic applications making use of the auricular muscles and their neural networks.

## Introduction

The auricle of humans and other mammals contains three extrinsic and six intrinsic muscles (1, 2). The extrinsic muscles are the posterior auricular muscle (PAM), superior auricular muscle (SAM), and anterior auricular muscle (AAM), whereas the intrinsic muscles are the helicis major (HMJM) and minor (HMNM), tragicus (TR), anti-tragicus (ATR), transverse auricular muscle (TAM), and oblique (OAM) muscles. These muscles have been considered vestigial in humans, though it has been suggested that during development in the womb they may exert forces on the cartilage and affect the shaping of the ear (2, 3). In postnatal humans, they are rarely under voluntary control (2, 4). However, the neural connections of the auricular muscles with the brainstem and other deep brain structures are intact (5–7), and these muscles are easily accessible for wearable neuroprosthetics. Hence, they have been used as targets for numerous existing and potential future neuroprosthetic applications, for the diagnosis and treatment of a large range of diseases and health conditions, including neurological disorders, brainstem injuries, emotional states, and auditory functions. They have also been used as a bio-controller for assistive devices.

The aims of this review are (1) to provide a detailed account of the neural networks controlling the extrinsic and intrinsic auricular muscles, (2) to summarize the diagnostic and therapeutic functions of these muscles as described in the current literature, and (3) to outline existing and potential future neuroprosthetic applications based on the auricular muscles and their neural networks.

### Innervation of the Auricular Muscles

**Figure 1:**
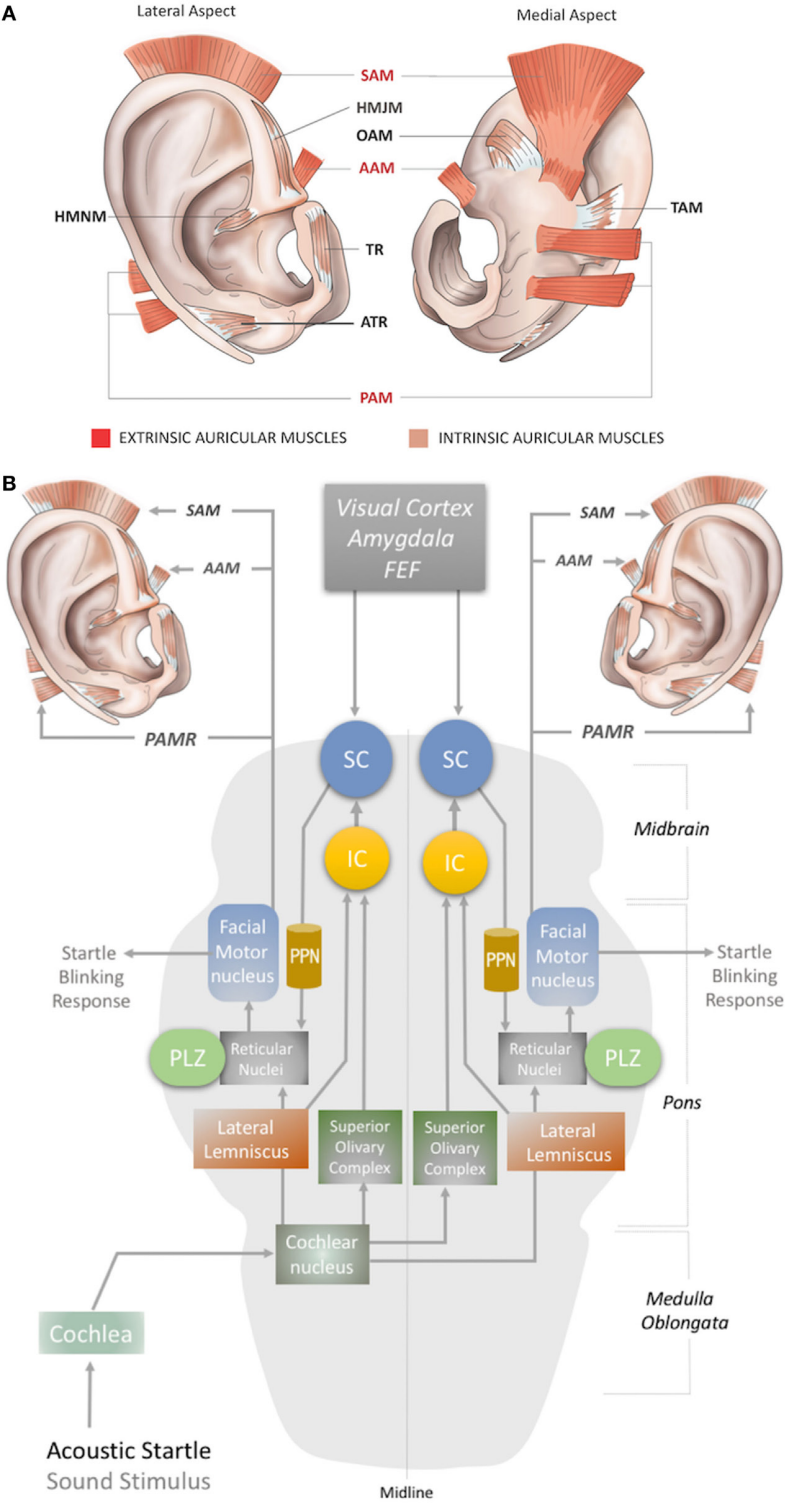
**(A)** Location of the extrinsic and intrinsic auricular muscles. The three extrinsic muscles (red shaded) are the superior auricular muscle (SAM), posterior auricular muscle (PAM), and anterior auricular muscle (AAM). The six intrinsic muscles (brown shaded) are the helicis major (HMJM) and helicis minor (HMNM) muscles, tragicus muscle (TR), anti-tragicus muscle (ATR), transverse auricular muscle (TAM), and oblique muscle (OAM). **(B)** The neural network of the acoustic PAMR reflex in the brainstem (11, 12). PPN, pedunculopontine nucleus; IC, inferior colliculus; SC, superior colliculus; FEF, frontal eye field; PLZ, paralemniscal zone.

In humans, three extrinsic auricular muscles—the SAM, AAM, and PAM—arise from the temporal aspect of the cranium and insert into the auricular cartilage (Figure 1A). They hold the auricles in place and are responsible for the reinforcement, positioning, and angle of the auricle (1, 8). They are innervated by the temporal (SAM and AAM) and PAM branches of the facial nerve (3, 9), and vascularized by the superficial temporal, posterior auricular, and occipital arteries (10).

The intrinsic auricular muscles have origins and insertions within the cartilaginous auricle (2). They play a role in the positioning and formation of the folds of the cartilaginous auricle by connecting the opposite margins of the fissures. Thus, the intrinsic auricular muscles contribute to the overall topography of the human ear. They also function as a sphincter of the external auditory meatus (3, 5, 8–13). The six intrinsic auricular muscles can be divided into two groups. The anterior group includes the HMJM, HMNM, TR, and ATR, and the posterior group includes the OAM and TAM (Figure 1A) (1). All the intrinsic auricular muscles are innervated by branches of the facial nerve and vascularized by branches of the superficial temporal, posterior auricular, and occipital arteries (1, 8). The temporalis branch of the facial nerve innervates the tragicus (TR and ATR) and helicis (HMJM and HMNM) muscles, but contributions of the posterior (HMNM) and inferior auricular (TR) branches of the facial nerve have also been reported (14–19). The TAM and OAM are innervated by the auricular-occipitalis branch of the facial nerve (14–21).

### Cortical and Subcortical Networks

**Figure 2:**
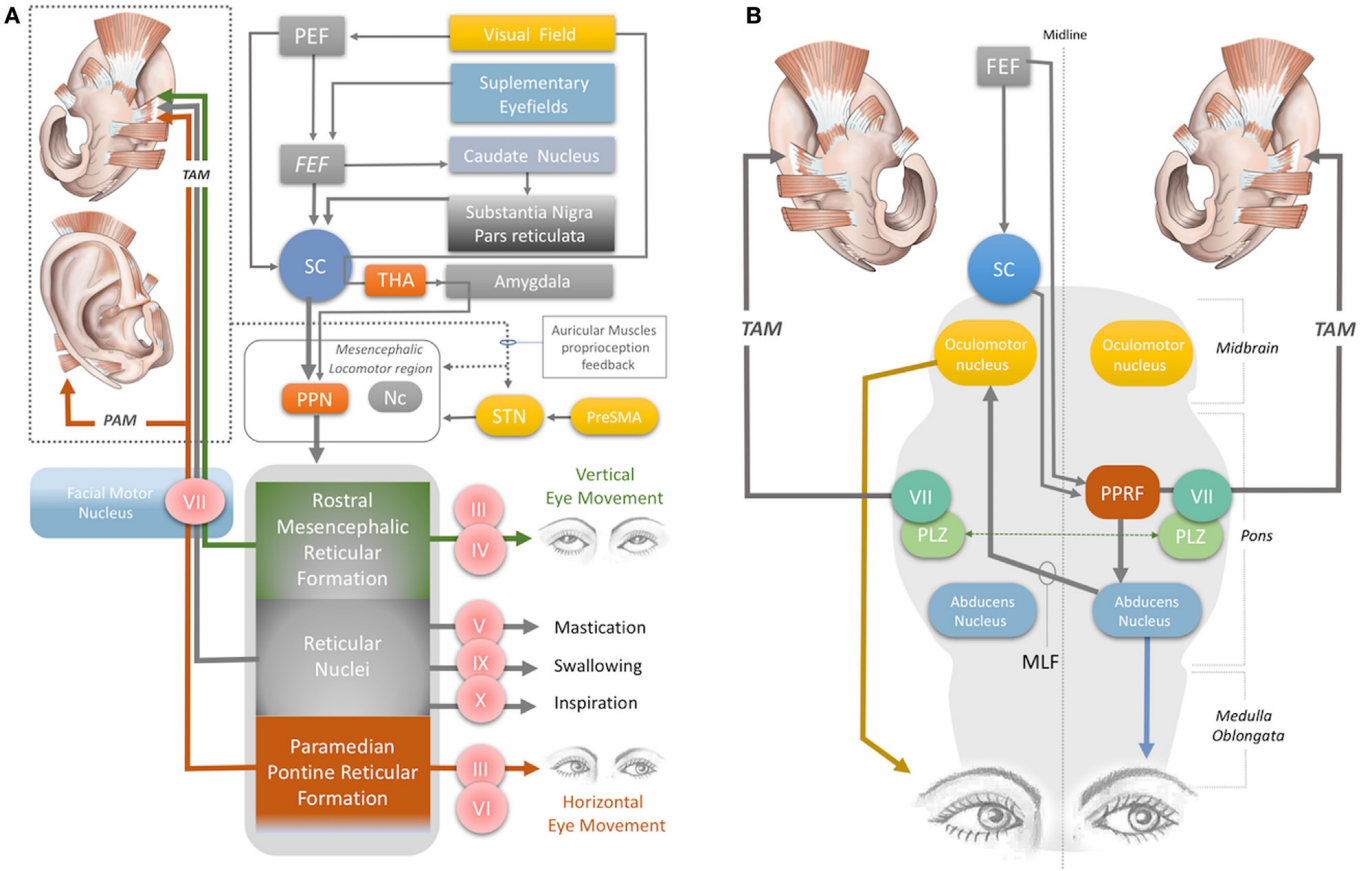
**(A)** Neural network acting on the auricular muscles, *via* the facial nerve and other cranial nerves. STN, subthalamic nucleus; PreSMA: pre-supplementary motor area; Nc, nucleus cuneatus; THA, thalamus; PEF, parietal eye field; FEF, frontal eye field; PPN, pedunculopontine nucleus; III, oculomotor nerve; IV, trochlear nerve; V, trigeminal nerve; VI, abducens nerve; VII, facial nerve; IX, glossopharyngeal nerve; X, vagus nerve. **(B)** Horizontal gaze and TAM coactivation networks—PPRF, paramedian pontine reticular formation; PLZ, paralemniscal zone; MLF, medial longitudinal fasciculus; VII, facial nerve motor nucleus; SC, superior colliculus; FEF, frontal eye field; TAM, transverse auricular muscle.

The auricular muscles have direct or indirect neural connections with a network that comprises brainstem structures and multiple cortical zones. At the brainstem level, the motor nucleus of the facial nerve contains motor neurons that directly control all the auricular muscles. In 1948, Szentágothai demonstrated that when the entire facial nucleus was lesioned, all three extrinsic auricular muscles showed 100% degeneration (22). In 1978, Schmidt and Thoden demonstrated that the cortical representation of the auricular muscles is closely associated with the region of cortex representing the frontalis and orbicularis oculi muscles (23). In 2001, Morecraft et al. investigated the projections to musculotopically defined subsections of the facial nucleus from the motor cortices, including the supplementary motor cortex and rostral cingulate cortex, in the rhesus monkey (24). Both cortical regions project bilaterally to the facial motor nucleus (25–27). The area of cortex that controls the upper facial muscles is innervated bilaterally by the facial subnuclei, while the lower facial muscles are innervated contralaterally by the facial subnuclei (28). Evidence for bilateral cortical control of the auricular muscles has been further consolidated by the following studies. It has been demonstrated that stimulation of area 8b of the frontal cortex [renamed later as the premotor ear-eye field] and Brodmann Area 9 are involved in both ear and eye movements (29–33). In addition, the supplementary eye fields (SEF), including the parietal eye field (PEF) in the parietal cortex, the frontal eye field (FEF), and the dorsolateral and medial prefrontal cortex, are reported to be involved in control of ear movements and transformation of auditory–visual sensory stimuli (34). However, microstimulation studies of the cortical ear motor control areas were unable to induce ipsilateral ear movements. Mainly bilateral responses were observed; only in specific subdivisional zones were contralateral responses elicited (7, 30). At the subcortical level, ear movements are controlled by the superior colliculus (SC) and its associated neural networks, including the inferior colliculus, reticular nucleus, and the motor nuclei of the cranial nerves, including the facial nerve (34–37) (Figures 1B and 2A).

## Existing and Potential Future Neuroprosthetics for Extrinsic Auricular Muscles

### Continuous Monitoring and Detection of Auditory Function, Intracranial Facial Nerve Palsy, and Brainstem Injury with the Aid of PAMR

Auditory stimuli such as clicks or tone-bursts can induce an electrical potential in the PAM in awake humans, which is referred to as the postauricular reflex (PAMR) (38–40). The muscle activity of the PAM in the PAMR can be measured using an EMG recording electrode placed on the posterior auricular skin superficial to the PAM (41). The PAMR produces a bilateral response even from a monoaural sound stimulus (42). Binaural stimulation causes a response that is equivalent in both amplitude and latency to the sum of the monaural responses (43). In this context, a unilateral design of a wearable ear neuroprosthetics might be convenient for continuous recording or monitoring of the PAM.

Although the precise circuity remains elusive, the proposed subcortical neural pathway underlying the PAMR includes the cochlea and cochlear nucleus as the first two steps, after which it splits into two pathways, through the superior olivary complex (bilaterally) and lateral lemniscus (Figure 1B). The pathway that conveys signals bilaterally to the superior olivary complex ultimately targets the inferior and superior colliculi. The SC connects to the reticular nucleus *via* the pedunculopontine nucleus (PPN). The reticular nucleus also receives the second pathway from the cochlear nucleus *via* the lateral lemniscus (Figure 1B). In the final link of the PAMR reflex, the paralemniscal zone (PLZ) and reticular nuclei activate the facial motor nucleus to contract the PAM (Figure 1B) (35–37, 44).

As the PAMR neural arc passes through the cochlea but not the vestibular system (45), it has been suggested as a useful basis for the diagnosis of auditory dysfunction in infants and children (46). Until recently, however, the PAMR has not been used routinely in the clinic, because of its variability within and across individuals (37). O’Beirne (44) improved the signal-to-noise ratio of the PAMR by placing a reference electrode on the pinna rather than the forehead and proposed an objective hearing test device using the PAMR (44). In this context, a design for a wearable device for PAMR monitoring, with an embedded acoustic stimulator, an EMG recorder for the PAM, and a ground electrode on the pinna can be conceptualized.

Another possibility for recording the acoustic PAMR is facial nerve monitoring. The facial nerve contains the efferent pathway (Figure 1B) that generates this response, as the reflex mechanism is seen on the ipsilateral side in individuals with intracranial facial nerve palsies (45). Thus, the PAMR could potentially be a tool for determining facial nerve conduction velocities. A biomedical device with a capability for bilateral EMG recording of the PAMR could help to determine the level of brainstem injury, as it would be affected by midline-crossing pathways (Figure 1B). As an antidromic approach, electrostimulation of the PAM might also help expedite neurorecovery of injured centers within the PAMR reflex pathway.

The magnitude of the auditory-evoked PAMR can be altered by eye rotation (45, 47). EMG activity in the PAM increases when the eyes rotate laterally, and thereby produces an enhancement of the PAMR (48) (Figure 2A). A complex neural network is responsible for this effect. The visual cortex and SEF, including the PEF and FEF, project to the SC *via* direct and indirect pathways, after which the SC activates the reticular formation *via* mesencephalic locomotor region structures, including the PPN (34, 49–52) (Figure 2A). The FEF and dorsolateral prefrontal cortex can also modulate the SC *via* a pathway through the caudate nucleus and the substantia nigra pars reticulata, and function as a secondary system to modulate the reticular formation (49–51) (Figure 2A). A somewhat simplified approach to the neural network architecture begins by splitting the reticular formation in the brainstem into subdivisions, including the rostral mesencephalic reticular formation (RMRF), paramedian pontine reticular formation (PPRF), and reticular nuclei. Stimulation of the reticular formation can activate motor cranial nerves in addition to the facial nerve motor nucleus (35–37). Each subdivision of the reticular formation has specific functions, and they project to the relevant cranial nerve motor nuclei (Figure 2A). The RMRF is responsible for vertical eye movements *via* the oculomotor and trochlear nerve motor nuclei (53, 54), whereas the PPRF is responsible mainly for horizontal eye movements *via* the oculomotor (contralateral) and abducens motor nuclei in the midbrain and pons, respectively (55, 56). Because the PAMR correlates with lateral gaze, in addition to facial nucleus and reticular formation (nuclei) connections, it can be postulated that the reticular formation sends signals to the SC and cortical ear–eye control centers to align the gaze and the ear to the same stimuli (Figure 2A). Therefore, the PAMR neural pathway can also be used as a proxy signal to determine the level and extent of a brainstem injury that might include the motor nucleus of oculomotor (midbrain), abducens (pons), and facial (pons) cranial nerves, if it is performed with and without lateral gazing. PAMR systems with integrated eye-tracking glasses could be developed for objective and quantitative assessments.

### Control of Assistive Devices with the PAM

The extrinsic auricular muscles can be used to produce signals to control assistive devices (57). This is particularly useful for individuals with neurological disorders such as quadriplegia, because the facial nerve that innervates these muscles is unlikely to be affected by high-level spinal lesions. In 1999, Friedman et al. found that the extrinsic auricular muscles can be activated voluntarily and can generate action potentials up to 680 pV in amplitude. They used PAM potentials to control a paddle in a computerized ping–pong task (57). Their finding is further supported by a recent study that presented a myoelectric auricular control system based on the activation of the PAM, allowing tetraplegic individuals to control wheelchairs (58). One of the limitations for using the PAM to control assistive devices, however, would be the influence of emotions on PAM activity.

### Monitoring Emotional States with PAM, SAM, and AAM

The PAMR can be used, along with the acoustic startle blink reflex, for monitoring the emotional states of patients (e.g., with post-traumatic stress disorder). The PAMR can be potentiated by pleasant pictures and inhibited by aversive pictures, particularly pictures that have high emotional intensity (59). Generally, the magnitude of the PAMR is larger during pleasant stimuli, including happy expressions, pictures, and sounds, whereas aversive stimuli, including angry faces, result in a smaller magnitude (60–62). This is the opposite of the modulatory effects on the startle blink reflex and suggests the potential value of the PAM for monitoring emotional state, as an adjunct to monitoring the acoustic startle blink reflex.

It has been demonstrated that the underlying neural network for influence of emotion on PAMR response involves mainly the visual pathway that conveys the emotional component of the visual stimulus to the amygdala *via* the SC and thalamus (63, 64). When the amygdala (e.g., the central nucleus) is stimulated, it acts on the reticular formation directly or *via* the PPN (63, 64) (Figure 2A). Consequently, it modulates facial muscle responses, including those of the auricular muscles.

In addition to the PAMR, the SAM and AAM may also show a reflex response as an effect of emotional modulation (65, 66). It is notable that these two muscles have different sizes than the PAM. The SAM is the largest of the three extrinsic auricular muscles, followed by the PAM, and then the AAM. The differences in size may provide a mean level of difference in the reflex magnitude, based on the area of the muscles (45). In principle, all the extrinsic auricular muscles could be targets for wearable emotion monitoring devices. Conversely, because none of them is free of emotional modulation, a better option for control of assistive devices would be the intrinsic auricular muscles such as the helicis major and minor; so far there is no report of emotional modulation of this group of intrinsic muscles.

## Intrinsic Auricular Muscles

### Detection of Brainstem Lesions: TAM

The oculo-auricular phenomenon is a bilateral coactivation of the TAM during lateral gaze of the eyes (67, 68). Coactivation of ear and eye muscles is common in mammals, as discussed previously. The SC is involved in this coactivity. Several studies have found that when the SC was electrically stimulated, contralateral gaze deviations and bilateral pinna movements took place (69–73). The SC, the contralateral PPRF (at the level of the pons), the ipsilateral oculomotor nerve in the midbrain, the contralateral abducens nerve in the pons, and the ipsilateral and contralateral facial nuclei are involved in the production of these movements, *via* their effects on interneurons in the ipsilateral PLZ (Figures 2A,B) (74–83). The levels of midline-crossing axons were reported to be at the pons (80) and midbrain (84). These extensive neural connections of the TAM allow its EMG signals to be used as a proxy to monitor the integrity of the relevant networks. A study of 1,186 patients with brainstem lesions demonstrated that the absence of TAM coactivation ipsilateral to a lateral gaze indicates supranuclear brainstem lesions, because the tracts decussate at the mid-pontine level. The lesions in these patients were found to be in the ipsilateral mid-pontine or contralateral midbrain areas (6) (Figure 2B).

The oculo-auricular phenomenon in the TAM may not be restricted to lateral gaze (and PPRF), but may also apply to vertical gaze. In 1978, Schmidt and Thoden reported that the TAM was coactivated (43%) with vertical eye movements; however, it is worth noting that this response was not confirmed by others (6, 23). Vertical eye movement is modulated by the midbrain vertical gaze center in the RMRF of the midbrain (53, 54) (Figure 2A). The RMRF stimulates the motor nuclei of the oculomotor and trochlear nerves to drive vertical eye movements (53, 54) (Figure 2A). Thus, EMG recordings of the TAM may be used to clarify the neural circuits in the midbrain, including the vertical gaze center in the RMRF and the two cranial nerve nuclei in the midbrain (trochlear and oculomotor nerve). However, the existence of an oculo-auricular phenomenon for the TAM with vertical gaze needs to be validated by further studies.

### Monitoring Stroke Manifestations (Swallowing, Mastication, Inspiration): TAM

Monitoring the TAM also has potential value for assessing stroke-related motor dysfunctions. Although it is innervated by the facial nerve, activation of the glossopharyngeal and vagal nerves during coughing, swallowing, and inspiration also results in activation of the TAM (23) (Figure 2A). The TAM is also influenced by mastication, indicating coactivation by the motor division of the trigeminal nerve (Figure 2A). Disorders in mastication, breathing, and swallowing (dysphagia) occur in up to 50% of stroke patients and may cause aspiration pneumonia or poor nutrition (85–88). EMG monitoring of the TAM with wearable devices may allow an opportunity to continuously monitor the status of all these disorders.

### Alleviation of Parkinson’s Symptoms: HMJM, HMNM, TR, and ATR

Recently, Cakmak et al. (89) stimulated the TR, ATR, and HMNM muscles with a wearable electrostimulator in a double-blind randomized clinical trial and demonstrated a clinically significant improvement in the motor symptoms of Parkinson’s disease. The proposed mechanism of action was the stimulation of the subthalamic nucleus (STN) and potentially the pre-supplementary motor area, reticular formation, and mesencephalic locomotor region, which includes the PPN and nucleus cuneatus (Figure 2A).

The upper part of the facial muscles shows bilateral hemispheric control by the facial nerve (90). It has been reported that unilateral STN stimulation induces bilateral motor-evoked potentials in the orbicularis oculi muscle in Parkinson’s patients (91). The intrinsic auricular muscles (TR and ATR) have also been shown to contract simultaneously with the orbicularis oculi muscles (5); this provides indirect evidence of bilateral cortical and STN connections to the intrinsic auricular muscles. Bilateral STN stimulation is a common modality of deep brain stimulation for alleviating Parkinson’s disease motor symptoms, especially non-axial symptoms. Axial symptoms, such as postural instability and gait difficulties, are related to the PPN (92, 93).

A recent fMRI study demonstrated that stimulation of the anti-tragicus muscle zone can activate the nucleus cuneatus, which receives proprioceptive input from the neck muscles (94). The nucleus cuneatus and the PPN are the two major components of the mesencephalic locomotor region, which modulates posture and gait (95) (Figure 2A). In this context, stimulation of the intrinsic auricular muscles could potentially modulate the mesencephalic locomotor region, thereby influencing posture and gait (Figure 2A).

## Conclusion

The extrinsic and intrinsic auricular muscles have extensive and intact neural connections within the brainstem, deep brain structures, and the cortex, including motor and limbic neural structures. Although the neural networks of the auricular muscles are not fully understood, this review provides an insight of their connections with neural networks to underline their existing and potential future use for the diagnostic and therapeutic devices.

## Author Contributions

Developed the concepts: YC; performed literature review: ML; performed advanced literature review: YC and MZ; data collection: YC and ML; analyzing the literature: YC, MZ and ML; neuronal network diagrams: YC; wrote the draft: ML; edit the draft: YC; wrote the main paper: YC, ML, and MZ.

## Conflict of Interest Statement

YC has one granted and three pending patents for wearable neuroprosthetics based on auricular muscles. The other authors declare no conflicts of interest. The reviewer ES and handling editor declared their shared affiliation.
